# Environmental modelling of climatic sensitivity and performance stability of semi-transparent photovoltaic systems in a tropical coastal region

**DOI:** 10.1038/s41598-026-58363-8

**Published:** 2026-06-22

**Authors:** Nuha Desi Anggraeni, István Seres, István Farkas

**Affiliations:** 1 Doctoral School of Engineering Sciences, Hungarian University of Agriculture and Life Sciences, Páter K. u. 1., Gödöllő, H-2100 Hungary; 2 Institute of Mathematics and Basic Science, Hungarian University of Agriculture and Life Sciences, Páter K. u. 1., Gödöllő, H-2100 Hungary; 3 Institute of Technology, Hungarian University of Agriculture and Life Sciences, Páter K. u. 1., Gödöllő, H-2100 Hungary; 4 Department of Mechanical Engineering, Institut Teknologi Nasional Bandung, Jl. PH. H. Mustofa No 23, Kota Bandung, 40124 Jawa Barat Indonesia

**Keywords:** Climate resilience, Environmental modelling, Energy yield, Performance ratio, Sustainable energy transition, Regional environmental change, Climate sciences, Energy science and technology, Environmental sciences

## Abstract

This study investigates the climatic sensitivity and long-term performance stability of a semi-transparent photovoltaic (STPV) system operating in a tropical coastal region of Indonesia. Using a decade of daily meteorological data (2012–2022), we developed a multivariate regression-based environmental modelling approach to evaluate the influence of key climatic variables on performance ratio (PR) and energy yield. Three modelling structures were considered, including a full-variable model, a simplified model based on global tilted irradiance (GTI) and ambient temperature, and a constant PR benchmark. The results indicate that GTI and temperature are the dominant climatic drivers, accounting for most of the meaningful variability in PR. The simplified GTI-temperature model achieved predictive performance comparable to the full model, suggesting that a parsimonious formulation can retain most of the explanatory power while reducing data requirements. The estimated PR values ranged between 0.78 and 0.80, consistent with reported values for tropical photovoltaic systems. Despite observable seasonal and interannual climatic variability, the system exhibited relatively stable performance over the study period, with no clear monotonic decline in energy yield. These findings highlight the applicability of simplified environmental models for performance assessment and planning in data-scarce tropical coastal regions.

## Introduction

The global transition towards sustainable energy has accelerated the deployment of photovoltaic (PV) technologies across diverse climatic zones^1,2^. Tropical coastal regions are highly vulnerable to the impacts of climate change, including sea-level rise, intensified storms, and altered precipitation patterns^3,4^. Concurrently, these areas offer substantial opportunities for solar energy development. Establishing resilient renewable energy infrastructure in these areas is essential for climate change adaptation and mitigation^5,6^. This study investigates the long-term performance of semi-transparent photovoltaics (STPV), a dual-use technology, as a sustainable energy solution in vulnerable contexts^7,8^.

STPV technologies represent an innovative solution at this energy-environment nexus. By allowing partial light transmission, STPV systems enable dual land-use functions, such as building-integrated photovoltaics (BIPV) and agrivoltaics, where energy production is synergistically integrated with agriculture or architecture^9–11^. The multifunctionality of this approach is especially significant in coastal tropical regions, characterised by intense land competition and an increasing demand for climate-adaptive infrastructure^12,13^. STPV can directly contribute to multiple Sustainable Development Goals (SDGs), specifically SDG 7 (affordable and clean energy), SDG 11 (sustainable cities and communities), and SDG 13 (climate action).

Despite their potential, the efficacy of STPV systems in tropical coastal environments remains inadequately investigated. These regions are defined by elevated relative humidity, frequent cloudiness, moderate to high wind velocities, and pronounced seasonal fluctuations in irradiance and temperature. Such conditions affect photovoltaic performance differently than in temperate regions, especially for STPV modules, which exhibit unique thermal and spectral responses compared with conventional silicon modules^14^.

**Fig. 1:**
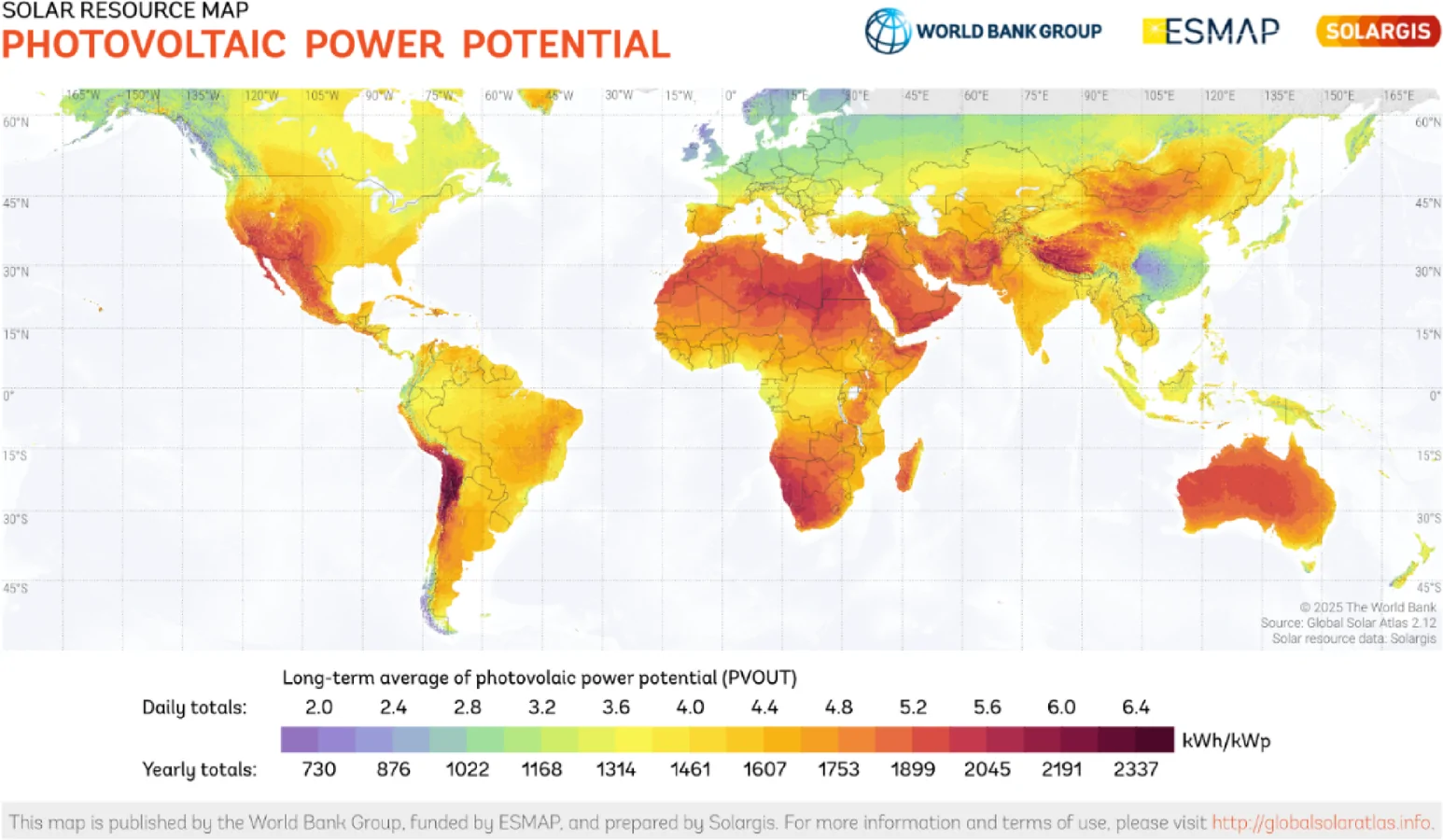
Global distribution of photovoltaic electricity output potential (PVOUT), expressed in kWh kWp⁻¹ year⁻¹, derived from the World Bank Global Solar Atlas, illustrating higher solar photovoltaic potential in equatorial and tropical regions^15^.

Climatic variability substantially affects the operational efficiency of PV modules, especially STPV systems, which exhibit distinct thermal and spectral responses compared with conventional opaque silicon modules. As illustrated in Fig. 1, the global distribution of photovoltaic power output potential (PVOUT) exhibits marked spatial variability. While many tropical regions retain substantial solar potential, the highest PVOUT values are generally observed in dry subtropical zones rather than humid equatorial regions. Nevertheless, tropical coastal environments remain important contexts for evaluating the climate-sensitive performance of PV systems under real operating conditions.

The performance ratio (PR) is a commonly utilised metric for evaluating photovoltaic efficiency, reflecting the impact of temperature, soiling, inverter losses, and environmental factors. Nonetheless, PR is frequently reduced to a constant or modified solely for temperature, potentially neglecting the cumulative impacts of humidity, wind, and irradiance fluctuations^16,17^. Long-term studies have shown that tropical climates can sustain PR values between 0.75 and 0.82^18,19^, yet no comprehensive analysis has been carried out for STPV systems in tropical coastal settings.

PR remains one of the most widely used indicators in photovoltaic assessment because it provides a normalised measure of system performance that facilitates comparison across locations and system configurations. However, PR also has important limitations. It compresses multiple loss mechanisms into a single metric, may obscure interactions among climatic effects, and is often treated as a constant or only temperature-corrected in practical evaluations. These limitations are particularly relevant in tropical coastal environments, where irradiance variability, humidity, and thermal effects may co-occur over seasonal and interannual timescales.

The novelty of this work lies in incorporating location-specific climatic data into performance forecasting, thereby providing a more accurate assessment of STPV feasibility in tropical off-grid environments. The study presents a streamlined yet precise regression model that harmonises computational efficiency with predictive accuracy, providing practical benefits for energy planners, designers, and policymakers in the Global South. These findings enhance system design, planning, and policy frameworks for climate-resilient solar energy technologies, directly advancing sustainability objectives and equitable energy access in at-risk coastal areas.

Recent studies in solar energy utilisation have progressively shifted from traditional rooftop assessments to more extensive, contextually sensitive frameworks. Previous research in tropical regions has mainly concentrated on solar resource evaluation and conventional photovoltaic performance, indicating that irradiance and temperature significantly influence system behaviour. In contrast, long-term performance ratios in tropical climates typically remain within a consistent range. Simultaneously, recent studies have expanded the subject by exploring building-integrated photovoltaic (BIPV) beyond rooftops, conducting multidimensional assessments of urban solar energy, and performing large-scale simulations of solar radiation on intricate building surfaces. These studies underscore the growing importance of high-resolution spatial analysis, multi-source data integration, and multidimensional evaluation frameworks for solar deployment in built environments.

Nonetheless, despite these advancements, the long-term climatic sensitivity evaluation of STPV systems in tropical coastal regions remains insufficiently addressed. Specifically, there remains insufficient evidence regarding the efficacy of simplified, low-data regression models in encapsulating the primary climatic determinants of STPV performance over extended observational durations in humid coastal environments. This gap is particularly significant as the current study analysed a decade of daily data (2012–2022) from Sekongkang, Indonesia (Latitude: −8.9914°, Longitude: 116.8675°), and assessed the comparative influences of GTI, ambient temperature, humidity, and wind on performance ratio and specific energy yield. To better position the present study within the existing literature, Table 1 summarises representative previous studies that have either focused on solar-resource assessment, urban building solar utilisation, or conventional PV performance evaluation. In contrast, the present study specifically addresses the long-term climatic sensitivity and performance stability of an STPV system in the tropical coastal region using a parsimonious regression-based framework.

**Table 1:** Comparison of representative studies related to solar energy.

Study	Context/location	System focus	Main approach	Main limitation for the present topics	Relevance to the present study
Quansah et al. (2022)^8^	Ghana	Solar radiation resource	Assessment of solar radiation resource from NASA-POWER reanalysis products	Focuses on solar-resource assessment rather than STPV performance modelling	Provides tropical solar-resource context
Susanto et al. (2022)^19^	Indonesia	Conventional solar panels	Performance analysis in a tropical region	Limited climatic-sensitivity modelling and no STPV focus	Supports comparison of PR behaviour in Indonesia
Saleheen et al. (2021)^18^	Malaysia	Grid-connected solar PV	Performance assessment and model development	Building-scale conventional PV; not STPV and not coastal tropical case-specific	Supports PR comparison and simplified modelling relevance
Saka (2024)^20^	Grid-connected PV/agrivoltaic context	PV power plant	Performance evaluation	Does not address the long-term climatic sensitivity of STPV	Demonstrates the broader relevance of performance evaluation
Ni et al. (2025)^21^	Urban/building surfaces	BIPV beyond rooftops	High-accuracy prediction using multi-source data, shading computation, and machine learning	Focuses on urban building-surface potential rather than climatic sensitivity of STPV performance	Highlights the recent expansion of BIPV research beyond rooftops
Ni et al. (2025)^22^	Hong Kong urban buildings	Urban solar energy	Multidimensional evaluation framework	Focuses on urban energy planning rather than long-term empirical performance modelling	Shows the importance of multidimensional solar-energy evaluation
Ni et al. (2023)^23^	Metropolitan building surfaces	Urban solar radiation assessment	Large-scale solar-radiation simulation framework	Focuses on solar-radiation simulation, not PR-based system behaviour	Serves as a methodological contrast to site-based system modelling
Yan et al. (2025)^24^	Xiamen, China	Urban solar potential	Cooling-demand-driven multidimensional assessment	Urban solar-potential study rather than empirical STPV performance modeling	Reinforces the need for context-specific solar assessment frameworks
Present study	Sekongkang, Indonesia; tropical coastal region	Semi-transparent photovoltaic (STPV)	Regression-based climatic-sensitivity modeling	Single-site case study	Addresses long-term climatic sensitivity and model parsimony for STPV in a tropical coastal environment

This work applies an environmental modelling perspective to assess the stability and climatic sensitivity of solar system performance under long-term coastal climate variability, rather than concentrating on component-level technology optimisation.

## Methodology

### Study area and environmental context

This study used meteorological and solar radiation data from Sekongkang, West Sumbawa, Indonesia (Latitude: −8.9914°, Longitude: 116.8675°). This location represents a typical tropical coastal zone in Southeast Asia, characterised by a consistent solar resource but also subject to regional climate change pressures, including changing monsoon patterns and coastal erosion^25^. This site selection facilitates the evaluation of STPV performance under optimal energy conditions and relevant environmental stressors.

### Data sources and collection

A daily meteorological dataset spanning from January 1, 2012, to December 31, 2022, was compiled. The variables included:

- Ambient temperature (°C).
- Atmospheric pressure (hPa).
- Relative humidity (%).
- Wind components (U and V vectors, m/s), from which wind speed (m/s) and direction (° from North) were derived.
- Global Tilted Irradiance (GTI, in Wh m^-^² and kWh m^-^²), obtained from validated satellite-derived products and transposition models that convert horizontal global irradiance to the plane of the optimally tilted STPV array.

#### Data pre-processing and analytical workflow

The daily meteorological dataset was screened for consistency before modelling. All variables were harmonised to a common daily temporal resolution to ensure comparability across the full study period (2012–2022). Derived wind metrics, including wind speed and wind direction, were calculated from the U and V vector components. Irradiance values were expressed at the optimally tilted surface to better represent the operating conditions of the STPV array.

**Fig. 2:**
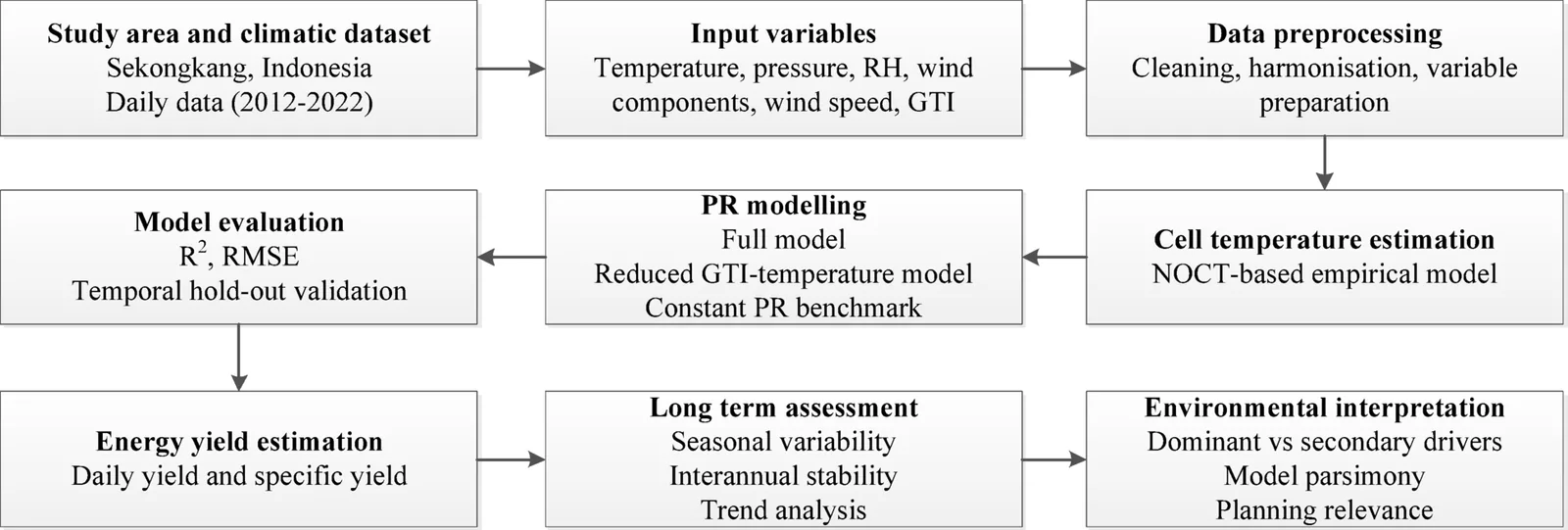
Technical workflow of the methodological framework adapted in this study.

The analytical workflow consisted of several sequential steps: First, the dataset was compiled and pre-processed to ensure consistency and suitability for modelling. Second, the photovoltaic cell temperature was estimated using an empirical NOCT-based formulation. Third, the performance ratio (PR) was modelled using three alternative structures: a full-variable regression model, a reduced GTI-temperature model, and a constant PR benchmark. Fourth, model performance was evaluated using standard statistical indicators. Fifth, the validated PR estimates were used to calculate daily and specific energy yield. Finally, the results were interpreted through long-term assessment of seasonal variability, interannual stability, trend behaviour, and their implications for environmental and climate-resilient planning. The overall methodological workflow is summarised in Fig. 2.

### Performance modelling and analysis

#### Cell temperature estimation

The photovoltaic cell temperature was estimated to account for thermal losses affecting module efficiency. A simplified linear model was used:

1 $$\:{T}_{cell}={T}_{ambient}+\left(\frac{GTI}{800}\right)\times\:\:\left(NOCT-20\right)$$

where:

T_cell_ is the estimated cell temperature (°C).

𝑇_ambient_ is the daily mean ambient temperature (°C).

GTI is the daily irradiance (Wh m^[-2^).

NOCT is the nominal operating cell temperature, assumed constant at 45 °C.

Equation (1) represents an NOCT-based empirical approximation commonly used in photovoltaic studies to estimate cell temperature from ambient temperature and irradiance conditions. Although simplified, this formulation is widely accepted for long-term system-level assessment where detailed thermal measurements are unavailable. This empirical model captures the increase in cell temperature when solar irradiance exceeds ambient levels.

#### Performance ratio (PR) modelling

To model the climatic sensitivity of the STPV system, a multivariate regression framework was developed for estimating the daily performance ratio (PR). Three modelling approaches were explored:

- *Full-variable regression model*: Incorporates all available climatic variables: ambient temperature, pressure, relative humidity, wind U and V components, wind speed, wind direction, and GTI.
- *Reduced-variable model*: Includes ambient temperature and GTI to test the explanatory power of the two most influential factors.
- *Constant PR model*: A baseline scenario assuming a fixed PR value of 0.799, consistent with conventional assumptions in tropical climates.

The general regression form used for the full model is:

2 $$\:PR={a}_{0}+{a}_{1\:}T+{a}_{2}\:P+{a}_{3}\:RH+{a}_{4}\:U+{a}_{5}\:V+{a}_{6}\:WS+{a}_{7}\:WD+{a}_{8}\:GTI+\epsilon\:$$

where:

T is ambient temperature (°C);

P is atmospheric pressure (Pa);

RH is relative humidity (%);

U, V are wind components (m s^[-1^);

WS is wind direction (m s^[-1^);

WD is wind direction (°).

GTI is global tilted irradiance (Wh m^[-2^).

*a*_*0*_, *a*_*1*_,*…*,*a*_*8*_, $\:\epsilon\:$ are regression coefficients.

Equation (2) does not represent a fixed industry-standard formula; rather, it is defined as a general form of multivariate linear regression model developed in this study to quantify the influence of climatic variables on daily PR. The linear structure was selected to prioritise interpretability, transparency, and consistency with the study’s environmental modelling objective. The models were trained and validated using historical data from 2012 to 2022. Variable importance and sensitivity were analysed using standardised regression coefficients and feature scaling.

#### Energy yield and sustainability indicators

The daily energy output of the STPV system was estimated by combining the PR model with irradiance input:

3 $$\:{E}_{daily}=PR\times\:\:GTI\times\:\:{P}_{STC}$$

where: $\:{E}_{daily}$ is daily energy yield (kWh); PR is estimated performance ratio; GTI is daily irradiance on the tilted surface (kWh m^[-2^); and P_STC_: Installed system capacity, assumed to be 3.3 kWp.

Equation (3) follows the standard logic for PV energy yield estimation by combining irradiance input, installed system capacity, and performance ratio to yield a system-level output relationship. This formulation provides a transparent basis for comparing long-term yield behaviour across varying climatic conditions. The yield was standardised to specific yield (kWh kWp^[-1^) to enable scalability and comparison. The assessment of system resilience involved analysing stability and variability in PR and specific yield over 11 years.

The modelling framework is deliberately structured to emphasise interpretability and applicability for regional-scale sustainability assessment, rather than focusing on component-level system optimisation. This method aligns with the study’s aim to facilitate climate-resilient energy planning in the face of environmental variability.

#### Environmental model rationale

This study intentionally uses a regression-based environmental modelling methodology rather than comprehensive physical or simulation-based methods. The aim is to create an interpretable and adaptable model that simplifies regional system-climate interactions rather than enhancing individual system components. These simplified models are particularly relevant for environmental assessment and policy evaluation, where data availability, transparency, and computational efficiency are essential constraints.

The adopted framework has several limitations. First, the analysis is based on a single-site case study, which limits the immediate generalisability of the findings. Second, the regression structure prioritises interpretability over physical detail and therefore does not explicitly capture nonlinear or component-level interactions. Third, the absence of module-level diagnostic data constrains direct assessment of degradation mechanisms. These limitations are acknowledged to maintain transparency regarding the scope and applicability of the modelling framework.

#### Model evaluation and validation

Model performance was evaluated using the coefficient of determination (R^2^) and the root mean square error (RMSE). These indicators were used to assess both the explanatory strength and the predictive deviation of the full-variable and reduced-variable regression models. To reduce the risk of overfitting and assess predictive robustness under unseen climatic conditions, a temporal hold-out validation strategy was adopted, in which models were calibrated on an earlier portion of the dataset and then evaluated on a later independent period. This approach was selected because the dataset is temporally structured and the objective of the study is long-term environmental assessment rather than purely descriptive fitting.

### Analysis of climate resilience and sustainability indicators

A review of pertinent Indonesian policy frameworks, including the National Energy Policy (RUEN) and the National Climate Change Adaptation Plan (RAN-API), was conducted to connect technical findings with regional sustainability. This analysis identifies potential entry points for integrating STPV technology into climate-resilient development planning.

## Results and discussion

The significance of STPV systems extends beyond technical performance; they may also serve as adaptive energy infrastructure in regions experiencing environmental change. In the present study, the climatic sensitivity and long-term stability of STPV performance were examined under tropical coastal conditions using a regression-based environmental-modelling framework.

### Long-term climatic trends and environmental profile

**Fig. 3:**
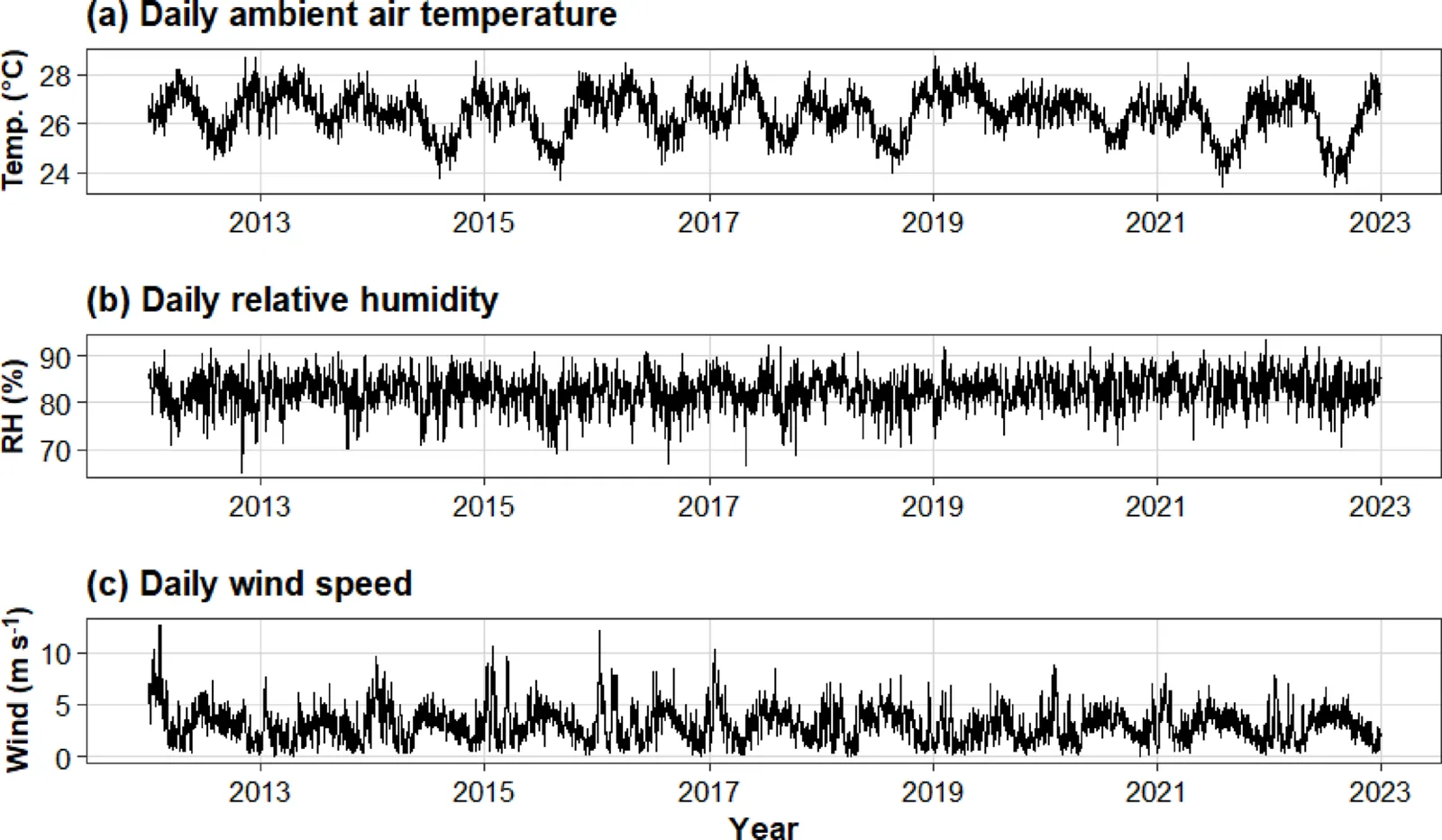
Long-term climatic profile of Sekongkang, West Sumbawa, Indonesia, during 2012–2022: **(a)** daily ambient temperature (°C), **(b)** daily relative humidity (%), and **(c)** daily wind speed (m s^[-1^.

Figure 3 presents the long-term climatic profile of the study area, including daily ambient air temperature (Fig. 3a), relative humidity (Fig. 3b), and wind speed (Fig. 3c) over the period 2012–2022. Ambient temperature remained within a relatively narrow range, approximately 23–29 °C, indicating limited seasonal thermal variability. Relative humidity was persistently high throughout the study period, mostly above 75%, consistent with the humid character of tropical coastal climates. Wind speed exhibited clearer short-term and seasonal fluctuations, with episode peaks and generally higher values during the dry season, suggesting a potentially relevant role in the convective cooling of photovoltaic modules.

These variables define the environmental context in which the STPV system operates and consequently act as boundary conditions for performance modelling. However, they should not be interpreted as the primary driver of energy generation, as photovoltaic output is primarily influenced by solar irradiance. At the same time, temperature, humidity, and wind primarily modulate conversion efficiency and thermal dynamics. The 11-year dataset verifies that Sekongkang maintains a relatively stable tropical coastal climate, although with observable interannual variability. The mean annual ambient temperature was approximately 26.2 °C, exhibiting minimal long-term fluctuation. Relative humidity remained consistently elevated across all years, indicating persistently moisture-rich atmospheric conditions. Wind conditions exhibited seasonal patterns, with stronger flow during the dry season, which likely enhanced natural ventilation around the PV modules.

From an environmental modelling standpoint, these climatic characteristics are significant as they define the long-term environmental background within which irradiance-driven system performance must be interpreted. The climatic patterns illustrated in Fig. 3 therefore provide the boundary conditions for the subsequent analysis, while the dominant forcing variable for energy generation is analysed independently through GTI in the following section.

### STPV performance and dominant climatic drivers

**Fig. 4:**
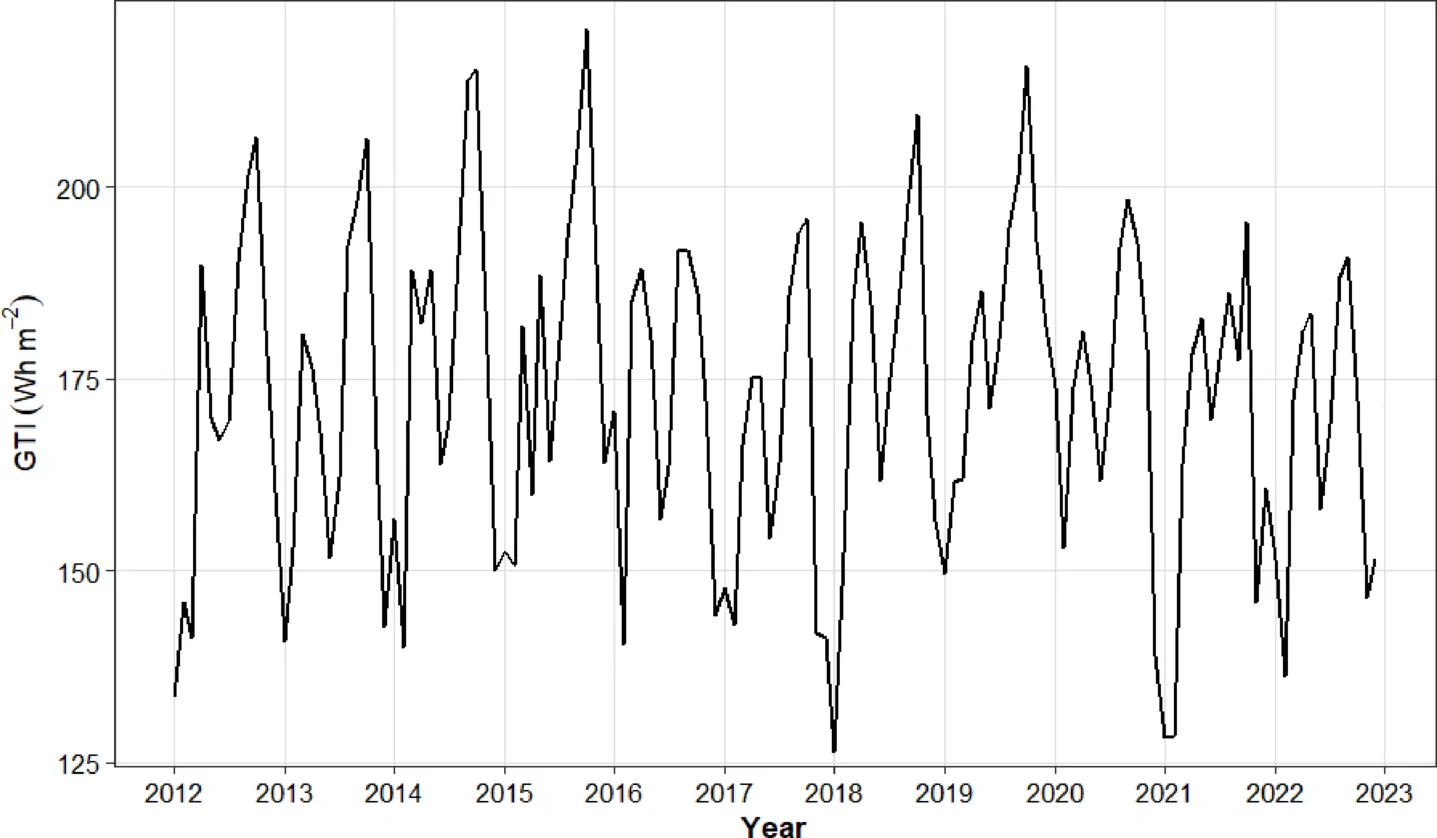
Daily global tilted irradiance (GTI), defined as solar irradiance incident on the optimally tilted photovoltaic surface, in Sekongkang, West Sumbawa, Indonesia, from 2012 to 2022.

Figure 4 displays the daily GTI in Sekongkang, derived from transposed horizontal irradiance data. GTI exhibits a clear seasonal pattern, with peak values observed during the dry season, particularly between August and October. Monthly average GTI ranged from approximately 133 (kWh m^− 2^) during low-resource months to values exceeding 200 (kWh m^− 2^) during the late dry season, confirming the strong seasonality of the solar resource. The irradiation pattern is consistent with the region’s monsoonal climate and provides the principal forcing for STPV energy production. In contrast to temperature, humidity, and wind, which act primarily through efficiency modulation, GTI directly governs the incoming solar resource available for conversion. For this reason, GTI is expected to dominate both variability and energy-yield behaviour.

From an environmental-modelling perspective, the observed interannual variability of GTI and ambient temperature represents the principal climatic control on photovoltaic performance in the studied area. Additional variables, such as relative humidity and wind speed, exhibit seasonal variation but only limited long-term explanatory importance. This separation between dominant and secondary climatic drivers provides a basis for model simplification without compromising the reliability of environmental assessment.

### Thermal performance and cell temperature dynamics

**Fig. 5:**
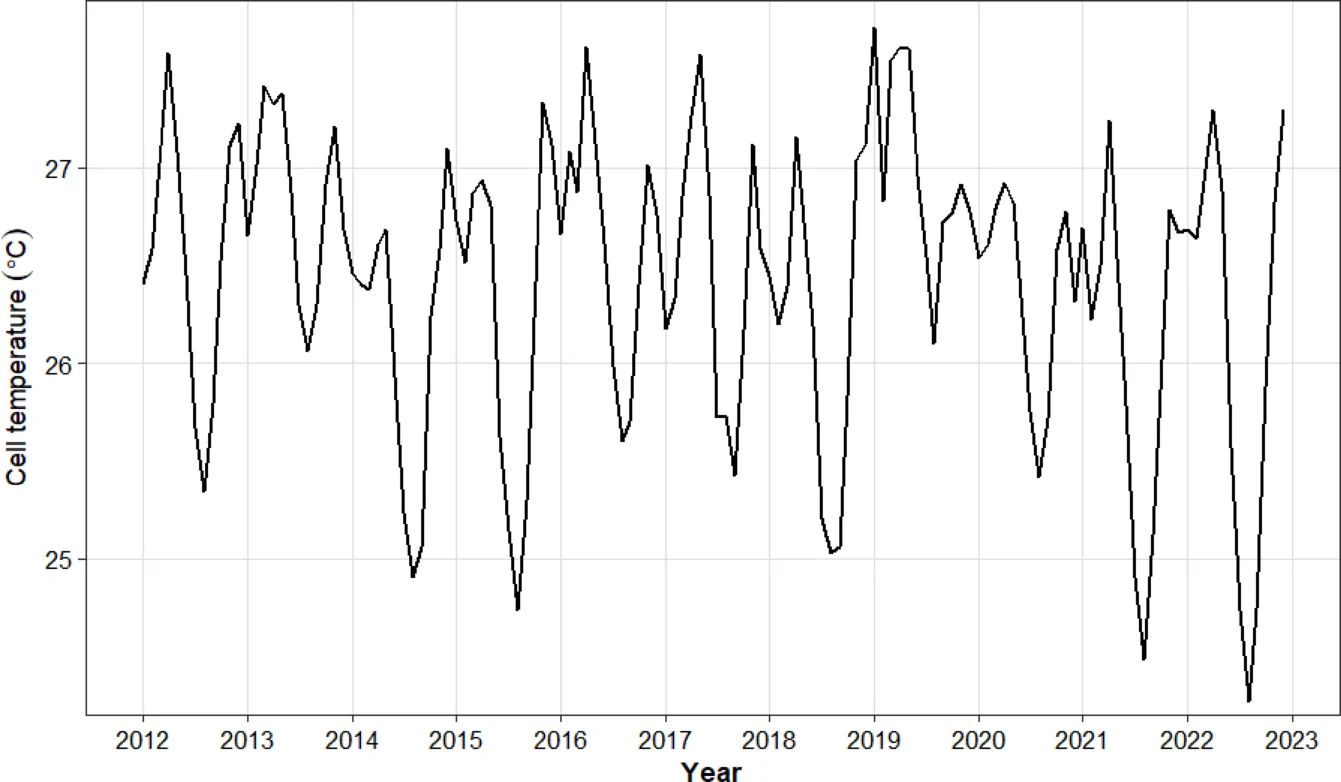
Monthly estimated photovoltaic cell temperature of the STPV system in Sekongkang, West Sumbawa, Indonesia, for the period 2012–2022, calculated using a NOCT-based empirical model.

The thermal behaviour of the STPV modules was evaluated using a NOCT-based cell-temperature estimation model. Figure 5 presents the estimated photovoltaic cell temperature over the study period. The estimated temperatures ranged from approximately 24.3 °C to 27.7 °C, with lower values generally recorded during the dry season and higher values during the wetter months. The seasonal behaviour of cell temperature reflects the interaction between solar irradiance and ambient temperature. Although irradiance peaks during the dry season, the simultaneous reduction in ambient temperature and stronger coastal winds appear to moderate excessive module heating. This suggests that thermal derating in Sekongkang is partially buffered by the local coastal environment, even during periods of elevated solar exposure.

The relatively limited seasonal temperature range indicates that the study site provides thermally stable conditions for STPV modules. This is relevant for environmental assessment because thermal stability reduces uncertainty in long-term performance projections and supports the use of simplified temperature-correction schemes in system-level modelling. While elevated cell temperature remains an important source of efficiency loss, the results suggest that temperature acts as secondary climatic control relative to irradiance in this tropical coastal setting.

### Performance ratio sensitivity to climatic variables

To assess the climatic sensitivity of the STPV system, PR was modelled using three alternative structures:

1. *Full-variable model*: Includes temperature, pressure, humidity, wind components, and GTI.

$$\begin{aligned}\:PR=0.925&-0.005\:T-{7.8\times\:10}^{-19\:}P-{6.3\times\:10}^{-20}\:RH+{1.67\times\:10}^{-18}\:U-{4.3\times\:10}^{-19}\:V\\&-{1.2\times\:10}^{-18}\:WS-{9.8\times\:10}^{-20}\:WD-0.00013\:GTI+0\end{aligned}$$

2. *Simplified model*: Includes only temperature and GTI.

* PR=0.925-0.005T-0.00013GTI*

3. *Constant PR benchmark*: Uses a fixed PR of 0.799.

The full model incorporated the available climatic variables, including temperature, pressure, relative humidity, wind components, wind speed, and GTI. The reduced model included only GTI and ambient temperature, whereas the benchmark assumed a fixed PR value representative of tropical PV applications. The regression results indicated that both the full and simplified models (GTI + temperature) performed comparably, with only minimal differences in predictive accuracy. The coefficient of determination (R²) surpassed 0.80 in both models, indicating that irradiance and temperature account for most of the PR variance in this tropical coastal setting. This trade-off is particularly important for environmental assessment, where model transparency, interpretability, and limited data requirements may be more valuable than small gains in statistical fit.

The estimated PR values range from 0.78 to 0.80, with a mean of approximately 0.792. This range aligns with performance ratios documented in comparable tropical environments, such as 0.75–0.80 in Indonesia^19^, and with long-term evaluations in Malaysia, which achieved PR values of 0.76–0.79^18^. Comparable ranges have also been observed in tropical Africa, where annual PR values typically range from 0.77 to 0.82^8^. These comparisons confirm that the observed PR values reflect tropical system performance and that the simplified GTI-temperature regression model serves as an effective forecasting tool for early-stage system planning^20^.

**Fig. 6:**
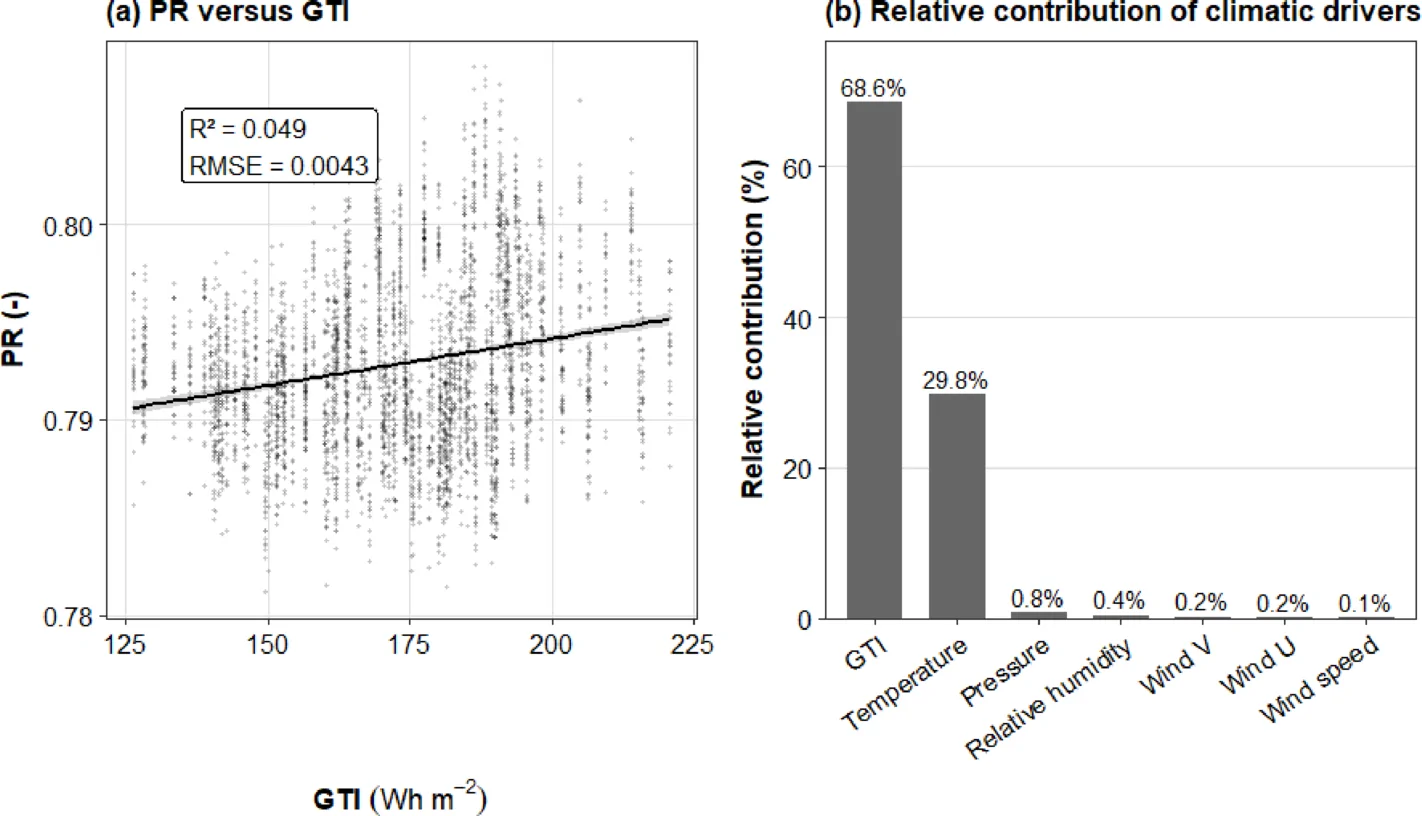
Relationship between the daily performance ratio (PR) of the semi-transparent photovoltaic (STPV) system and global tilted irradiance (GTI), showing a positive linear correlation between system performance and solar resource availability.

Figure 6 summarises the climatic sensitivity of PR in two complementary ways. Figure 6a shows the bivariate relationship between PR and GTI and indicates a weak positive relationship (R^2^ = 0.049; RMSE = 0.0043). This indicates that although higher irradiance is generally associated with slightly higher PR values, GTI alone does not fully explain PR variability. Figure 6b provides a more informative multi-variable interpretation by showing the relative contribution of each climatic predictor in the full regression model. GTI contributes 68.6% of the total standardised explanatory effect, followed by ambient temperature at 29.8%, while pressure (0.8%), relative humidity (0.4%), wind V (0.2%), wind U (0.2%), and wind speed (0.1%) make only marginal contributions. The dominance of GTI and ambient temperature indicates that these two variables account for nearly all of the meaningful climatic influence on PR in the study area. This hierarchical pattern supports the use of a parsimonious GTI-temperature model for simplified environmental assessment and practical forecasting. The present findings are broadly consistent with previous tropical PV studies, in which irradiance and temperature are commonly identified as the primary controls of performance. However, the weaker contribution of humidity and wind speed observed here appears more pronounced than in some earlier assessments. This difference may reflect the specific coastal setting of Sekongkang, the daily aggregation of the dataset, and the system-level regression framework adopted in the present study.

Although seasonal and interannual climatic variability is evident in the forcing variables, the PR values remain in a relatively narrow range. This indicates a stable system response to changing environmental conditions and strengthens confidence in the use of simplified models for long-term environmental assessment in data-scarce tropical coastal regions.

### Energy yield stability and climate resilience

**Fig. 7:**
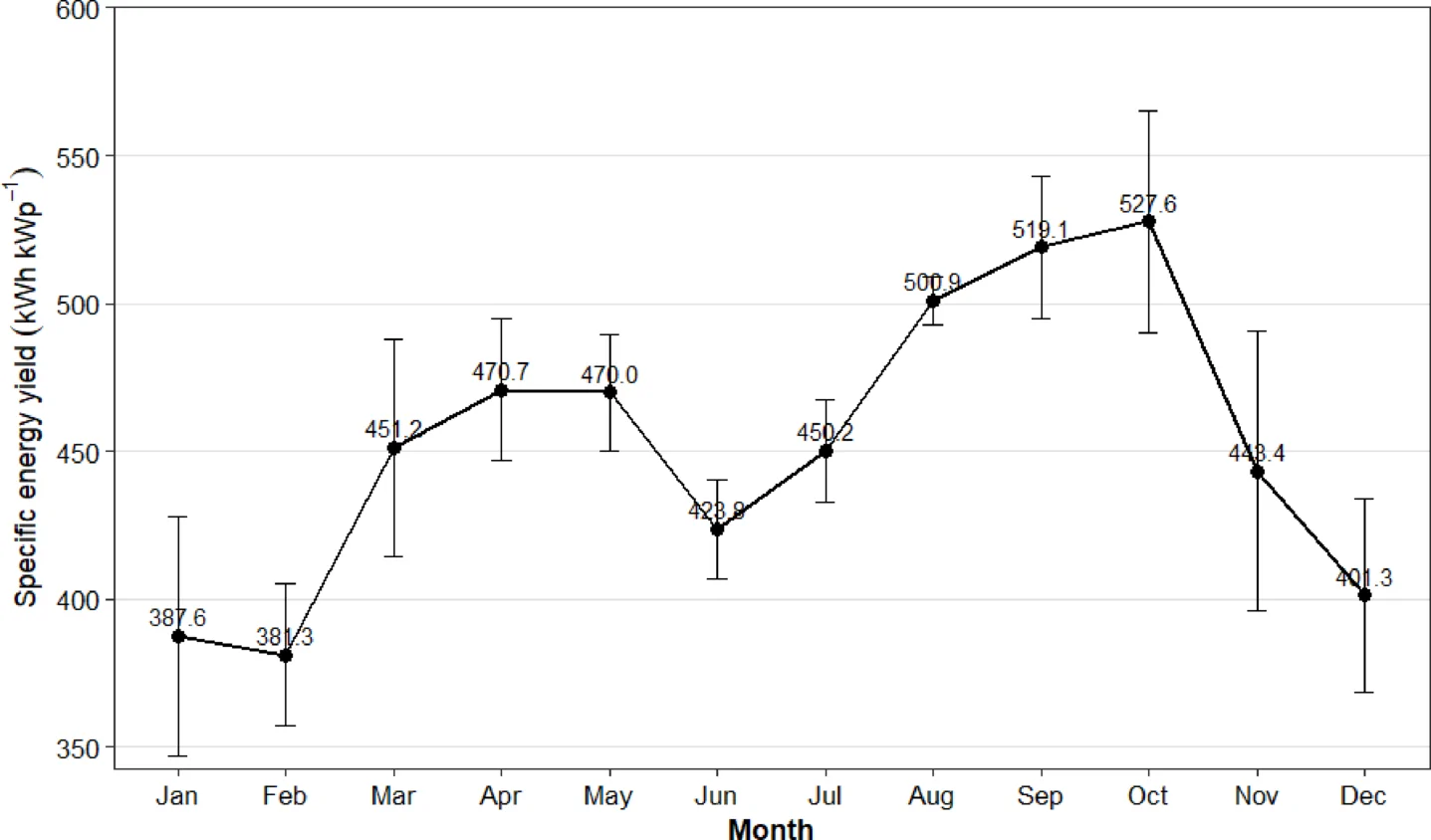
Monthly specific energy yield (kWh kWp⁻¹) of the 3.3 kWp semi-transparent photovoltaic (STPV) system in Sekongkang, West Sumbawa, Indonesia, averaged over the period 2012–2022.

Figure 7 presents the monthly-average specific energy yield of the 3.3 kWp STPV system in Sekongkang, averaged over 2012–2022, along with the interannual variability for each month. Monthly specific yields range from approximately 115 to 160 kWh kWp^− 1^ month^− 1^, with the highest mean values occurring during the late dry season, particularly from August to October, and the lowest values during the wetter months, especially around February.

The results indicate a clear seasonal performance pattern, primarily influenced by solar resource availability, with negligible thermal derating attributed to the stabilising effects of coastal winds. The significant rise in yield during the mid-to-late dry season corresponds with GTI peaks and comparatively lower humidity, highlighting the climate sensitivity of system performance.

The system demonstrates consistent high energy productivity throughout the year, supporting its applicability as a stable energy component for regional-scale planning in tropical coastal environments. The normalised values are a scalable reference for comparing system designs or performing feasibility analyses in similar geographic contexts^26,27^.

**Fig. 8:**
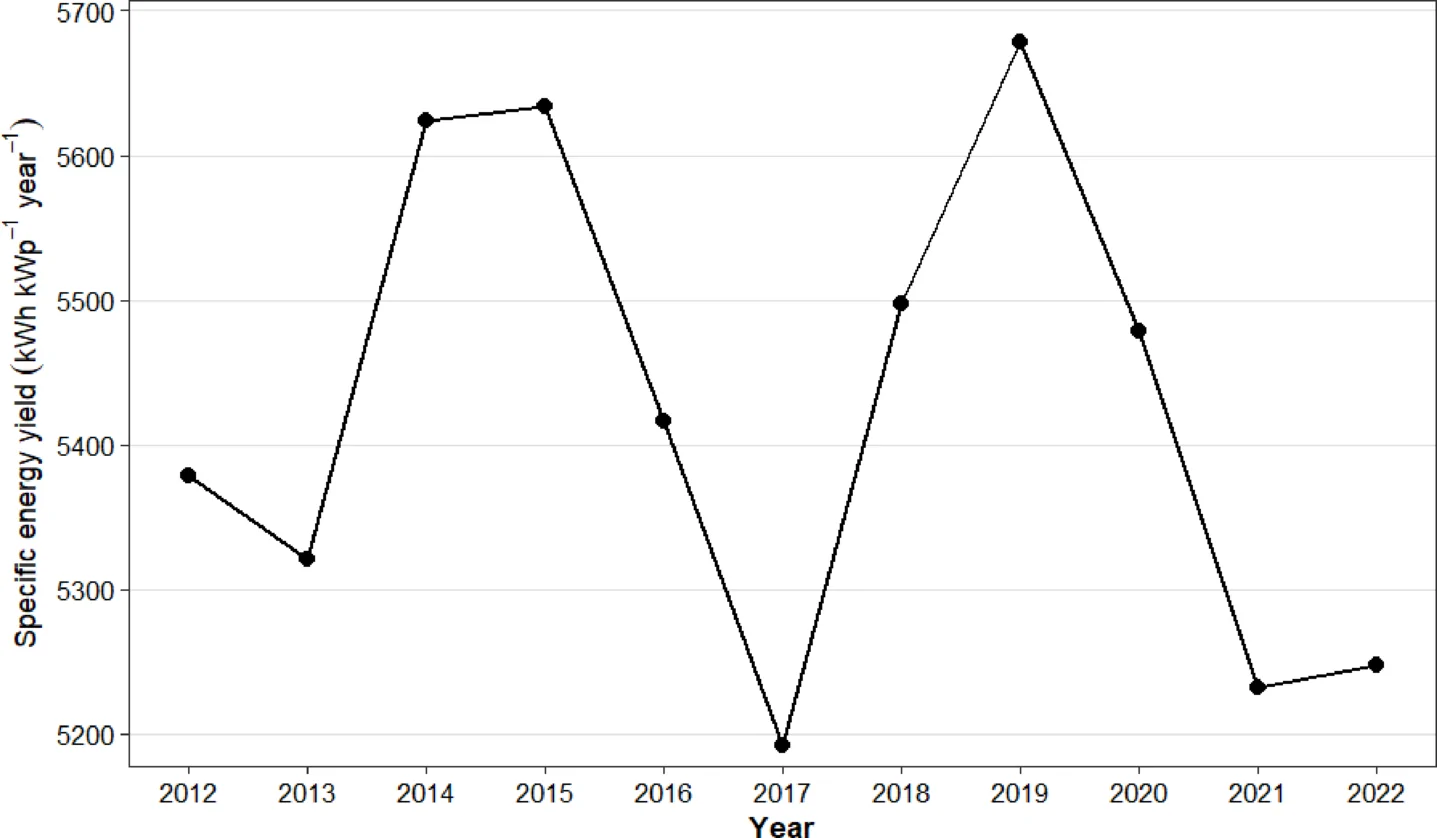
Annual specific energy yield (kWh kWp⁻¹ year^-1^ of the 3.3 kWp of STPV system in Sekongkang, West Sumbawa, Indonesia, from 2012 to 2022.

Figure 8 presents the annual specific energy yield of the 3.3 kWp STPV system in Sekongkang over 11 years from 2012 to 2022. The annual yield varies from 1,380 to 1,450 kWh kWp^− 1^, with an average of approximately 1,420 kWh kWp^− 1^ year^− 1^. The low interannual variability (< 5%) indicates the stability of solar resource availability in tropical coastal regions and the consistent thermal-electrical performance of the STPV system under varying environmental conditions. The annual stability observed here is relevant for environmental assessment and regional planning because it implies that long-term performance projections can be made with relatively limited uncertainty. Compared with previous studies on tropical PV systems, the yield stability observed in Sekongkang falls within the expected range for high-resource environments. Still, it is notable for its low interannual variability.

**Fig. 9:**
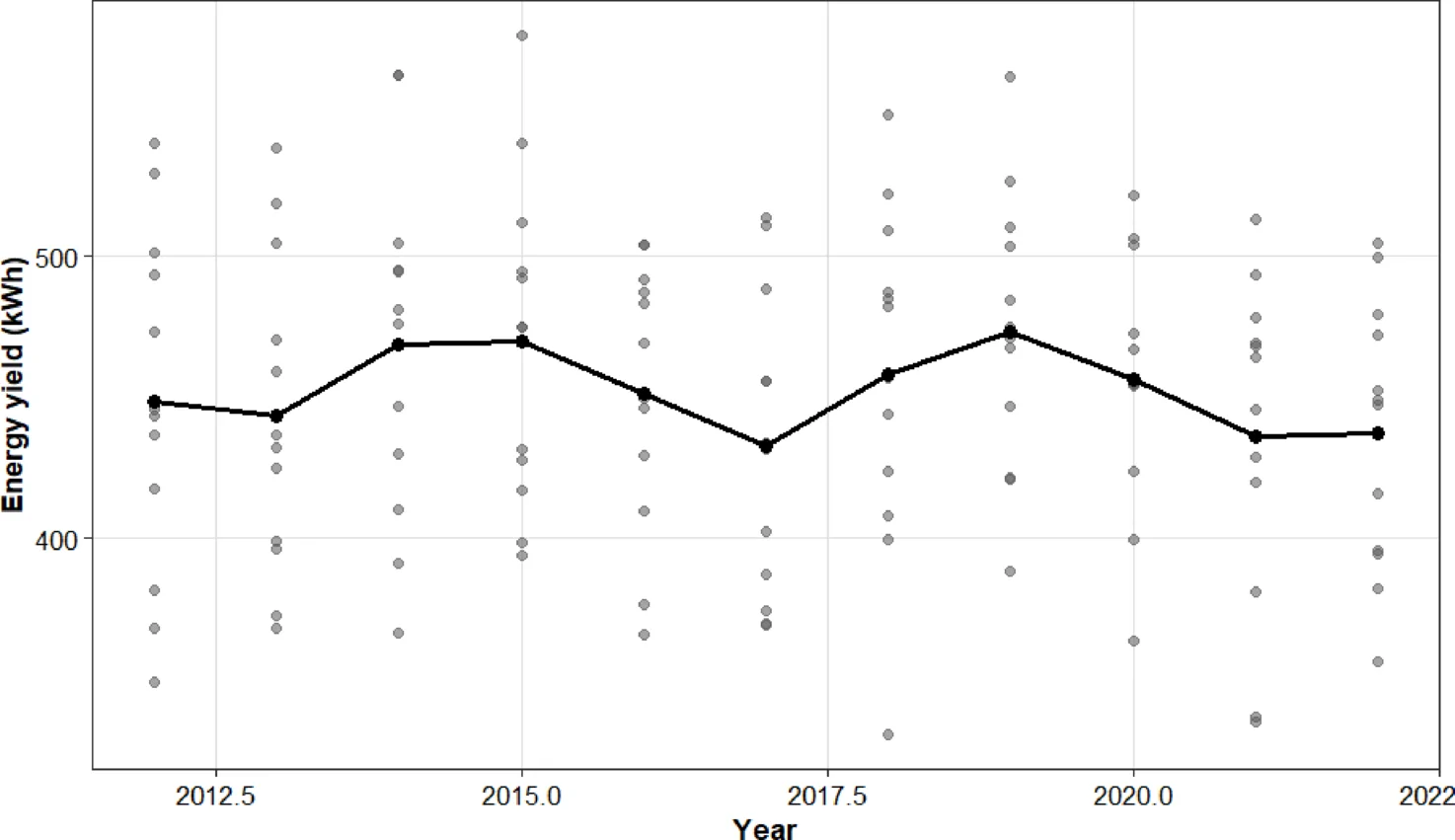
Linear trend of monthly energy yield of the semi-transparent photovoltaic (STPV) system in Sekongkang, West Sumbawa, Indonesia, from 2012 to 2022, indicating negligible long-term performance degradation.

Figure 9 shows the temporal distribution of monthly energy yield over the study period. No clear monotonic decline is visually apparent, and the yearly mean levels remain broadly stable. This suggests that no statistically significant degradation signal is detectable in the aggregated yield series over the study period. However, this result should be interpreted cautiously. The inference is based on a single-site case study and does not substitute for module-level degradation diagnostics. Interannual anomalies, including favourable climatic conditions in individual years, may also influence the apparent trend. Within the scope of this assessment, the long-term consistency of specific energy yield reinforces the suitability of simplified environmental models for regional-scale planning and long-term performance evaluation. Such consistency enhances confidence in climate-resilient energy planning in tropical coastal regions where detailed monitoring infrastructure is often unavailable.

### Implications for climate-resilience energy planning and sustainability

Beyond site-specific findings, the results demonstrate how simplified environmental models can inform climate-resilient energy planning under data-limited and climatically uncertain conditions. The results have significant implications for sustainable development and climate adaptation in tropical coastal areas.

i. Technological resilience: The insensitivity of STPV performance to high humidity and variable wind speeds is a significant advantage for coastal deployment, as traditional infrastructure is often subject to corrosion and degradation from moisture-related factors.

ii. Systemic interaction with adaptive land use: The consistent energy output, along with the technology’s semi-transparency, facilitates its incorporation into agrivoltaic systems. The dual-use approach enhances land-use efficiency, offers microclimatic benefits for specific crops, and diversifies income sources for coastal communities, thus contributing to the development of socio-ecological resilience^11^.

iii. Policy and planning utility: The validated simplified performance model serves as a low-data, high-utility instrument for regional energy planners and policymakers. This approach enables a swift evaluation of STPV’s potential in comparable coastal regions and facilitates incorporating these evaluations into Local Adaptation Plans and green recovery strategies.

Contributions to Sustainable Development Goals (SDGs): STPV systems provide reliable, clean electricity (SDG 7), facilitate sustainable land use (SDG 15), and offer infrastructure resilient to climate stressors (SDG 13), thereby serving as a practical technology for the implementation of integrated sustainable development pathways in vulnerable regions.

## Conclusions

This study presents a comprehensive, data-informed evaluation of the climatic sensitivity and sustainability performance of a semi-transparent photovoltaic (STPV) system in Sekongkang, a tropical coastal area in Indonesia. We analyse a decade of high-resolution meteorological and performance data (2012–2022) to present empirical evidence regarding the resilience and dual-use potential of STPV technology in an environment characterised by high humidity, seasonal variability, and increasing climate vulnerability.

The key findings are as follows:

1. Dominant climatic drivers: System performance is mainly determined by solar irradiance (GTI) and, to a lesser degree, ambient temperature. The two variables account for more than 80% of the variance in the daily Performance Ratio (PR). High relative humidity and variable wind speed, characteristic of the coastal tropics, were shown to have a statistically insignificant effect on efficiency, suggesting a built-in resilience to these environmental stressors.

2. The effectiveness of parsimonious models: a simplified predictive model that utilises only GTI and temperature, achieving accuracy comparable to that of a comprehensive multi-variable model. This tool is valuable for preliminary feasibility studies and energy planning in data-scarce coastal regions of the Global South, where rapid and reliable assessment is essential.

3. Demonstrated climate resilience: The STPV system demonstrated significant resilience, achieving a mean performance ratio of 0.792 over 11 years, with annual specific yields averaging approximately 1,420 kWh kWp^[-1^ year^[-1^. The long-term yield analysis did not reveal a statistically significant decline in aggregated performance over the study period. However, this result should be interpreted cautiously, as it is based on a single-site assessment and does not replace module-level degradation analysis. Future work should therefore combine long-term climatic modelling with component-level monitoring, multi-site validation, and broader sustainability assessment.

4. Sustainability and policy relevance: The semi-transparency of the modules enables dual-use applications, particularly in agrivoltaic systems, which can simultaneously promote energy (SDG 7) and food security (SDG 2) on constrained land. The results confirm that STPV is a technology aligned with climate-resilient development pathways. We advocate for its explicit incorporation into regional climate adaptation plans (e.g., RAN-API) and sustainable energy policies to facilitate integrated planning in vulnerable coastal areas.

This study employed comprehensive satellite-derived and modelled climate data; however, the importance of future long-term in-situ monitoring to assess microclimatic effects and validate degradation rates remains. Additional interdisciplinary research is necessary to quantify the socio-economic benefits and comprehensive life-cycle sustainability of STPV deployment in coastal communities.

In summary, this work establishes STPV technology as a reliable, resilient, and multifunctional component of sustainable energy transitions in tropical coastal regions. By bridging technical performance analysis with regional environmental change and policy discourse, we provide a framework for integrating climate-adaptive solar solutions into the broader agenda of equitable and resilient development.

## Data Availability

No datasets were generated or analysed during the current study.

## Abbreviations

STPV: Semi–transparent photovoltaic
PV: Photovoltaic
PR: Performance ratio
GTI: Global tilted irradiance
NOCT: Nominal operating cell temperature
PVOUT: Photovoltaic power output potential
BIPV: Building–integrated photovoltaic
SDGs: Sustainable Development Goals
RUEN: National energy policy (Indonesia)
RAN-API: National Climate Adaption Plan (Indonesia)
